# The Importance of Philanthropy Foundation for the Future Sustainability of Agriculture and Nutrition: An Opinion Study on Practical Applications, Policies, and Strategies

**DOI:** 10.3390/nu16081119

**Published:** 2024-04-10

**Authors:** Fahrul Nurkolis, Jodi Visnu, Nindy Sabrina, Hardinsyah Hardinsyah, Nurpudji Astuti Taslim, William Ben Gunawan, Melvin Junior Tanner, Nelly Mayulu, Mohammad Adib Khumaidi, Rony Abdi Syahputra, Mochammad Rizal, Raymond Rubianto Tjandrawinata, Trina Ekawati Tallei, Ray Wagiu Basrowi, Tonny Sundjaya, Lluis Serra-Majem

**Affiliations:** 1 Department of Biological Sciences, Faculty of Sciences and Technology, State Islamic University of Sunan Kalijaga, Yogyakarta 55281, Indonesia; fahrul.nurkolis.mail@gmail.com; 2 Marketing Strategy Consultant and Hospital Representative, Public Health Consultant and Health Educator, Panti Rapih Hospital, Yogyakarta 55223, Indonesia; jodi.c@mail.ugm.ac.id; 3 The Center for Health Policy and Management, Faculty of Medicine, Public Health and Nursing, Universitas Gadjah Mada, Yogyakarta 55281, Indonesia; 4 Nutrition Program, Faculty of Food Technology and Health, Sahid University of Jakarta, South Jakarta 12870, Indonesia; 5 Applied Nutrition Division, Department of Community Nutrition, Faculty of Human Ecology, IPB University, Bogor 16680, Indonesia; 6 Clinical Nutrition, Faculty of Medicine, Hasanuddin University, Makassar 90245, Indonesia; 7 Department of Nutrition Science, Faculty of Medicine, Diponegoro University, Semarang 50275, Indonesia; 8 Nutrition Coaching Development, PT. Prima Sehat Makmur Utama, Jakarta 12430, Indonesia; 9 Department of Nutrition, Faculty of Medicine, Universitas Muhammadiyah Manado, Manado 95249, Indonesia; 10 Faculty of Medicine and Health, Universitas Muhammadiyah Jakarta, Jakarta 15419, Indonesia; 11 Department of Pharmacology, Faculty of Pharmacy, Universitas Sumatera Utara, Medan 20155, Indonesia; 12 Division of Nutritional Sciences, Cornell University, Ithaca, NY 14850, USA; 13 Dexa Laboratories of Biomolecular Science, Dexa Medica Group, Cikarang 17530, Indonesia; 14 Department of Biotechnology, Faculty of Biotechnology, Atma Jaya Catholic University of Indonesia, Jakarta 12930, Indonesia; 15 Department of Biology, Faculty of Mathematics and Natural Sciences, Universitas Sam Ratulangi, Manado 95115, Indonesia; 16 Department of Community Medicine, Faculty of Medicine, Universitas Indonesia, Jakarta 10320, Indonesia; 17 Danone Specialized Nutrition, Jakarta 12940, Indonesia; 18 Department of Epidemiology, Faculty of Public Health, Universitas Indonesia, Jakarta 16424, Indonesia; 19 Centro de Investigación Biomédica en Red Fisiopatología de la Obesidad y la Nutrición (CIBEROBN), Institute of Health Carlos III, 28029 Madrid, Spain; 20 Research Institute of Biomedical and Health Sciences (IUIBS), University of Las Palmas de Gran Canaria, 35001 Las Palmas, Spain

**Keywords:** philanthropy, sustainable agriculture and nutrition, sustainability, foundation, future foods, climate action, nutrition and sustainable development, food charity

## Abstract

Food security, food sustainability, and malnutrition represent critical global challenges. Th urgency of comprehensive action is evident in the need for research collaboration between the food industry, agriculture, public health, and nutrition. This article highlights the role of philanthropy, of a non-profit organization, in supporting research and development and filling financial gaps. The article also explores the interplay of nutrition, agriculture, and government and policy, positioning philanthropy as a catalyst for transformative change and advocating for collaborative efforts to comprehensively address global food challenges. In addition, the discussion also underscores the ethical complexities surrounding charitable food aid, especially in terms of the dignity and autonomy of its recipients. The paper concludes by proposing future directions and implications, advocating for diversified intervention portfolios and collaborative efforts involving governments, businesses, and local communities. Apart from that, the importance of answering and alleviating ethical dilemmas related to food charity assistance needs to be a concern for future studies related to philanthropy because of the significant challenges faced by the contemporary food system, which include food security, health, and nutritional sustainability.

## 1. Introduction

Everyone concerned has to be aware of the significant diet-based public health concerns and the strain that growing populations, shifting diets, and shifting surroundings are putting on the food system [1]. Awareness of one challenge regarding how to meet human food demand despite the growing population still needs to be raised. This also accounts for climate volatility, water and energy availability, and soil conservation [1,2]. According to the United Nations, 2020 bore witness to a staggering 811 million individuals worldwide struggling with undernourishment, while a staggering 3 billion people found themselves unable to afford a healthy diet [3]. These issues underscore the pressing need for comprehensive action, particularly at the research level, where the food business, agriculture, public health, and nutrition all collaborate to address related issues. Discussing the main problems of food sustainability includes the production, consumption, and socio-economic challenges [4]. It has been proposed that investing in health technologies and health services to slow down population growth is a feasible approach [5]. Meanwhile, the transition to sustainable agriculture still lacks funding [6], even though this funding is the main factor in achieving sustainable agriculture and nutrition and implementing research, innovation, and new systems.

The food industry has a conservative business model with an easy-to-enter barrier, resulting in high competition in this industry. Therefore, innovation and creativity in research and development become distinctive features between companies. However, funding research and development—especially from the initial to the final stages—requires a hefty amount of funds and possibly a more extended payback period (break-even point). Moreover, this condition may be propagated by the fact that some companies will not focus fully on profit but on charity and corporate social responsibility as well, producing several challenges to investors and venture capitalists. With this in mind, we focused our attention on philanthropy. Several articles have highlighted the successful role of philanthropy in improving community health and advancing state-wide health [7,8,9]. Similar to the development of medical therapies, philanthropy may support the initial funding of research and development (RnD) stages, as they fill the critical funding gaps, as opposed to other ventures [10].

Despite these promising attributes, the specific mechanisms through which philanthropic organizations can address the intricacies of nutrition and food security still need to be explored. The significance of collaborative efforts involving charitable foundations, governments, businesses, and civil society in achieving sustainable agriculture and nutrition warrants further deliberation. Moreover, the ethical complexities surrounding charitable food aid as a focal point in the discourse on addressing food insecurity also need to be discussed. Therefore, this opinion article aims to articulate the practical implications of philanthropy’s potential to impact nutrition and, by extension, contribute to the sustainability of agriculture and food. Additionally, it endeavors to offer insights into the challenges and opportunities that lie on the horizon and provide recommendations to guide future research and actions.

## 2. Systematic Search Strategy and Selection Criteria of the Literature Study

References for this study were identified systematically via PubMed searches for articles published up to March 2024 using the terms ‘Philanthropy’ and ‘Nutriion’ and ‘Food Charity’ and ‘Agriculture’ and ‘Food Sustainability’ and ‘Agricultural Sustainability’. Additionally, relevant articles in this topic area were found through searches of Google Scholar and the Scopus Collection. Only articles published in English and the references cited within them were reviewed.

## 3. Philanthropy

**Figure 1:**
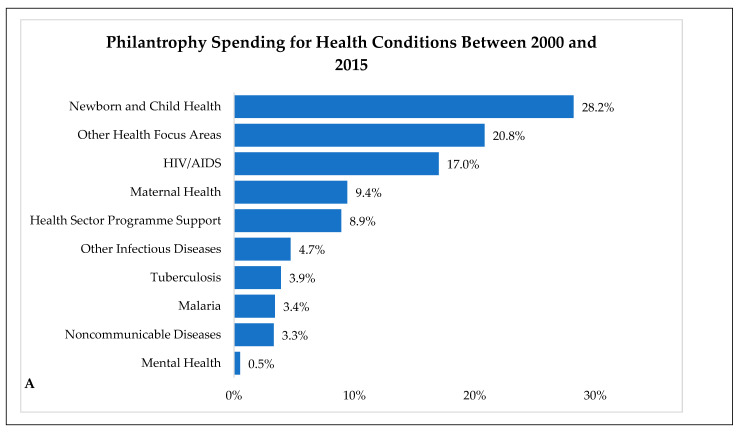

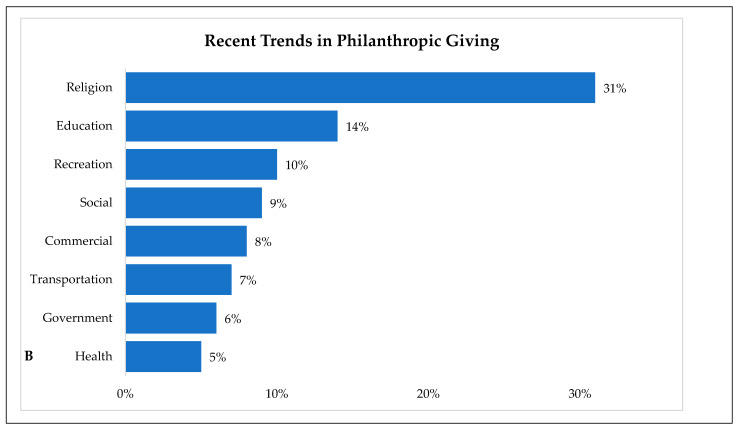
Percentage of Philanthropic Roles and Trends. (**A**) Philanthropy Spending for Health Conditions Between 2000 and 2015 (data obtained from https://unitedgmh.org and accessed on 24 January 2024). (**B**) Recent Trends in Philanthropic Giving (data obtained from https://store.givingusa.org/ and accessed on 24 January 2024). This graph was created referring to data provided by the related website which has been previously accessed and processed using GraphPad 10.1.1 Premium License by Fahrul Nurkolis.

A standard definition of philanthropy was a private gift made for the public good out of compassion, self-interest, or reciprocity; in other words, *voluntary action for the public good* [11]. The basis of philanthropic endeavors is the act of donation, which is associated with the objective of improving social determinants of health [12]. Philanthropy is highlighted as a different act of kindness or charity than helping family and friends since every act should have non-profit, sustainable, and voluntary principles (Figure 1) [13]. Furthermore, philanthropy has shown its capability and contribution to negotiation and the creation of policy (Figure 1) [14].

Scientific research financing is necessary to make the world a better place by creating more responsive science that advances knowledge for the benefit of humanity. Science-based philanthropy is undergoing significant growth along with its goal to solve problems such as diseases [15]. Aside from incentivizing new behaviors, health-related philanthropy also pays long-term “dividends” [10]. However, these philanthropies still face some novel challenges, such as their readiness to take on today’s perplexing problems, how to pinpoint uncharted research territories and use cutting-edge technology for discovery, how to maximize funding through innovative collaboration, how to address societal injustices, and how to involve the general public in research design [15].

Furthermore, the current challenge for philanthropy is related to the ethical implications of food philanthropy. Escajedo San-Epifanio and Rebato Ochoa (2022) clearly reveal that food security is nothing new and that the production and defense of a safe and stable food supply is also shrouded in ethical dilemmas [16]. Additionally, they also highlight that there are widely recognized structural injustices in relation to food, and it appears we still have a very long way to go to eliminate them, and that the human right to sustainable food must be a transversal and policy priority in future philanthropy settings. A new dilemma in food security is food redistribution by donors. Some food donors do not donate their food with the recipient in mind, but are more motivated by rewards. This can be answered by philanthropy, where philanthropy is designed to “*only give, don’t think about rewards*” because it is a grant. Apart from that, it requires an independent foundation to channel funds from philanthropists and not mention who the recipient will be. This might reduce the dilemma, and become a systemic challenge within food systems [17].

## 4. The Sustainability of Agriculture and Nutrition

The majority of the world’s poor, who are also most susceptible to disease and malnutrition, rely on food and agriculture as their primary source of livelihood. Food and agriculture growth can significantly contribute to reducing malnutrition and the resulting ill health [18]. The link between food, nutrition, and agriculture has been established as agricultural interventions have produced positive results on nutrition status determinants [19]. Moreover, nutrition-sensitive agriculture and sustainable development goals (SDGs) have been introduced as a critical approach to realizing food security in the field of nutrition [20]. According to the study presented at Think 20 (T20) Argentina 2018—which primarily discussed global governance and policy recommendations—the primary issue was how to get the necessary funds to support investments in sustainable food and agriculture systems, nutrition-related technologies, and the relevant infrastructure at the scale required to have a significant global impact [21].

**Figure 2:**
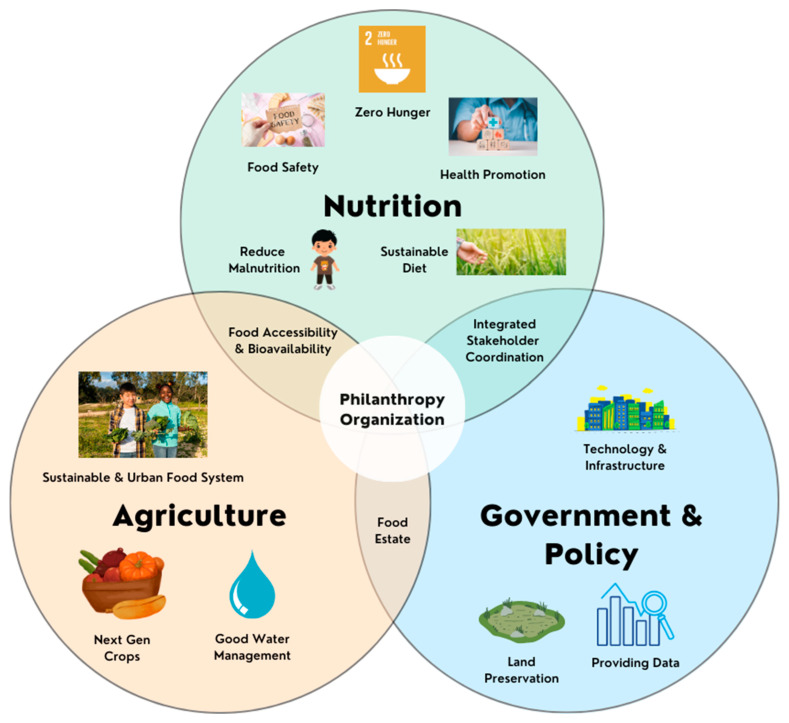
The potential contribution of philanthropic organizations to strengthening the sustainability of agriculture and nutrition. Created with BioRender.com Premium and Canva License by Fahrul Nurkolis.

Critical challenges in food, nutrition, and agriculture encompass soil health, sustainable water management, food diversity, cultural heritage, next-generation crops, urban food systems, and the health–agriculture nexus. McKenzie and William (2015) argued that, to creating sustainable agriculture, we must limit agricultural expansion, encourage new crops and greater genetic diversity, and focus on integrated farming systems and alternative energy sources for agriculture. By increasing the productivity of the existing cropland, the environment and climate change will be minimally impacted. Aside from production, food distribution must also be considered to solve hunger and famine [22]. Related to this matter, philanthropists can have many opportunities to improve quality of life, especially in nutrition and agriculture. One study highlighted the role of social finance, which utilized donations to cover the USD 2.5 trillion annual average global SDGs investment gap. The study recommends focusing on the establishment and funding of state-of-the-art research laboratories, ending hunger, improving nutrition, and achieving food security as the primary areas for fund utilization in the short and medium term [23]. Meanwhile, expanding quality agricultural land can be the best alternative to achieve the long-term target, i.e., sustainable agriculture. The general idea of the interplay between agriculture, nutrition, and governmental policies that can be mediated or initiated by philanthropy organizations is presented in Figure 2.

Climate change is a critical issue with far-reaching impacts on humanity, particularly exacerbating water scarcity and water-related disaster risks such as floods and droughts, along with major health problems, stemming from the disruption of the overall water cycle and significant changes in rainfall patterns [24,25,26]. Waterborne diseases claim more lives annually than all forms of violence or warfare combined. Indeed, infants and young children are more likely to die from waterborne diseases due to poor water sanitation than from conflicts or violence [27]. Therefore, the interconnection between water issues and climate change is inseparable in terms of enhancing human health, nutrition, and the sustainability of agriculture. Water sustainability refers to the maintenance and availability of clean water that can continue to fuel future generations for consumption, health, agricultural processes, and biodiversity, contributing to improved nutrition. However, our water supply faces its own set of challenges. The agricultural industry currently stands as the largest consumer of freshwater for both irrigation and livestock, paralleling the market demand for livestock products [28]. Water sustainability, focusing on the maintenance and availability of clean water, is vital for future consumption, agricultural processes, and biodiversity. Despite water being the most abundant natural resource on Earth, it is finite and irreplaceable. Therefore, maintaining water sustainability is crucial for human well-being, marine conservation, nutrition, agriculture, and future socio-economic development. Water is key to enhancing global health and productivity—meaning water sustainability is also key to the sustainability of agriculture and nutrition. The need for philanthropic funding programs to carry out water conservation practices not only helps combat the climate crisis but also significantly strengthens the socio-economic system. Sustainable water systems and practices must ensure that water can meet consistent needs without sacrificing the availability and quality of water for future generations through philanthropic programs. Ideal sustainability practices should aim to achieve three main goals: viability, accessibility, and environmental responsibility [29], all feasible through philanthropic donor funding with clear and straightforward regulations, thus achieving good water management.

However, while philanthropic efforts in addressing sustainability issues are pivotal, they face significant critiques in their ability to drive the systemic changes essential for true sustainability. As highlighted by Kraak and Niewolny (2024), philanthropic initiatives often fall short in addressing underlying systemic challenges within food systems [30]. One significant critique of philanthropic efforts lies in their tendency to address symptomatic issues within food systems rather than confronting underlying systemic challenges. This approach risks offering short-term solutions that fail to address the root causes of unsustainability. Moreover, while philanthropy provides crucial financial resources, it may not consistently advocate for the transformative changes needed in policies, practices, and governance structures to foster sustainability. Another critique revolves around power dynamics within philanthropic organizations, which often wield substantial influence in shaping agendas and priorities within the food system sector. This concentration of power can sideline the voices of local communities and grassroots organizations crucial for driving systemic change, potentially perpetuating dependency on external funding sources rather than fostering self-reliance and equitable resource distribution. Furthermore, the fragmented nature of philanthropic efforts can lead to the duplication of projects, lack of coordination, and inefficiencies. A more collaborative and coordinated approach is essential to maximize impact and drive systemic change. Additionally, philanthropic organizations may not always be held accountable for the outcomes of their investments in food systems, highlighting the need for greater transparency, evaluation, and accountability mechanisms. In light of these critiques, it is imperative for philanthropic efforts to reassess their approaches and align more closely with the transformative changes required for true sustainability in food systems. Collaboration, community engagement, long-term vision, and a focus on addressing root causes are essential for philanthropy to effectively catalyze meaningful and lasting transformation in food systems.

## 5. Unveiling the Impact of Philanthropic Initiatives

Figure 2 depicts a holistic view of sustainable agriculture and nutrition, highlighting the interconnectedness of three key domains: nutrition, agriculture, and government and policy. The true transformative power of philanthropy lies in the interplay between these circles. Within each circle, philanthropy plays a unique and multifaceted role, acting as a catalyst for positive change and a bridge between these domains. By supporting nutrition research, influencing agricultural practices, and advocating for enabling policies, philanthropic organizations create a synergistic effect that accelerates progress toward a sustainable future. This interconnected approach recognizes that addressing malnutrition requires a holistic understanding of the food system and a collaborative effort across all sectors. Meanwhile, Table 1 below provides a glimpse into the diverse efforts spearheaded by philanthropic organizations across the food system spectrum.

### 5.1. Nurturing Nutrition: A Philanthropic Focus on Health and Well-Being

At the heart of the diagram lies nutrition, representing the ultimate goal of a sustainable food system. Malnutrition remains a prevalent global challenge, with millions facing nutrient deficiencies and related health issues [31]. Philanthropy steps in as a champion for improved nutrition, focusing on interventions that promote healthy eating habits, increase access to nutritious food, and address underlying determinants of malnutrition. HarvestPlus is one of the philanthropic examples in this circle. Beyond quenching hunger, HarvestPlus’s biofortification efforts tackle the hidden hunger of micronutrient deficiencies. For instance, their work with orange-fleshed sweet potatoes stands as a testament to their success, demonstrably enhancing vitamin A intake in children [32,33,34,35]. This intervention is a critical step towards improving the immune system and reducing the burden of diarrhea, the second leading cause of death of young children in low- and middle-income countries [36]. Philanthropy is crucial in nurturing nutritional well-being by supporting research, empowering communities, and shaping policies prioritizing human health and dietary diversity.

### 5.2. Cultivating a Sustainable Agricultural Landscape: Philanthropy’s Seeds of Change

The agriculture circle represents the fundamental production system that underpins food security. However, conventional agricultural practices often face challenges like soil degradation, climate change, and resource depletion. Philanthropy emerges as a critical partner in fostering a sustainable agricultural future, supporting practices that protect the environment, enhance productivity, and improve farmer livelihoods. Philanthropic efforts in this domain include the Gates Foundation. Their support for developing drought-resistant maize varieties in Zimbabwe is a powerful illustration, enabling farmers to weather climate challenges and increase yields and strengthening food security for communities [37]. Philanthropy in agriculture cultivates a future where food production thrives in harmony with the environment, empowering farmers and fostering resilient food systems.

### 5.3. Bridging the Policy Gap: Philanthropy’s Advocacy for Change

**Figure 3:**
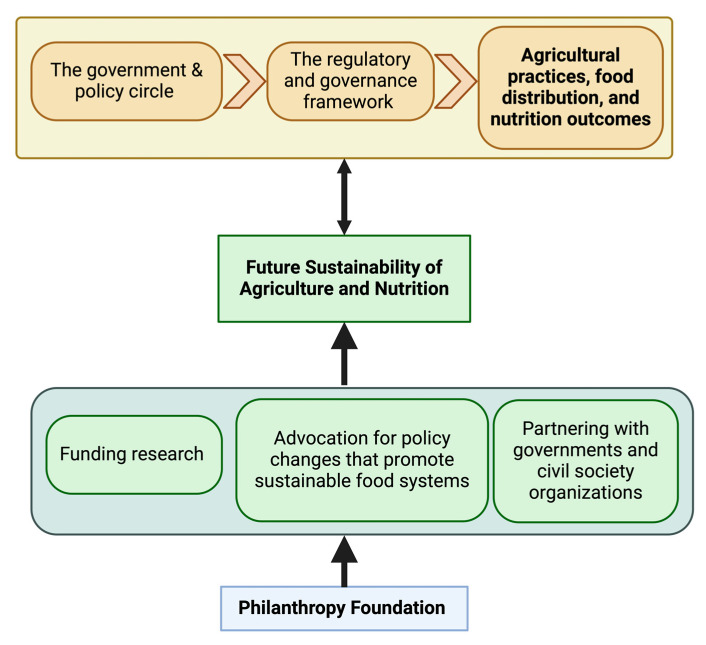
Keyways philanthropy influences government and policy.

The government and policy circle signifies the regulatory and governance framework that shapes agricultural practices, food distribution, and nutrition outcomes (Figure 3). Philanthropy, however, is not merely a beneficiary of these policies; it actively shapes them, bridging the gap between research, public needs, and legislative action (Figure 3).

Key ways in which philanthropy influences policy include:

1. Funding research that informs evidence-based policy formulation and providing data and insights that guide government officials in crafting effective regulations and programs (Figure 3);
2. Advocacy for policy changes that promote sustainable food systems, such as carbon pricing mechanisms or subsidies for organic farming practices, influencing legislative agendas towards environmental and health priorities (Figure 3);
3. Partnering with governments and civil society organizations to implement and monitor policies, ensuring effective translation of policy goals into tangible outcomes (Figure 3).

Philanthropy in the policy sphere acts as a bridge, fostering informed decision-making, advocating for progressive legislation, and ensuring the successful implementation of policies that advance sustainable food systems.

These are just a few examples highlighting the multifaceted impact of philanthropic initiatives across the food system. By analyzing these and countless others, we can learn valuable lessons, identify best practices, and collectively forge a path toward a future where nutritious food is accessible and sustainable food systems thrive.

## 6. Future Direction and Implications

Building food systems that, by the associated SDGs, promote climate resilience and environmental sustainability, protect biodiversity, and produce healthy diets for all is necessary to achieve a sustainable food future (Figure 2 and Figure 3). Institutional, technical, and financial breakthroughs are required to create such food systems and policies. Venture philanthropists have been crucial in shaping a new food and global health narrative. It was suggested that conducting impartial analyses of national and international philanthropic partnerships would enable us to examine cost–benefit ratios, sustainability standards, conflict of interest provisions, and the long-term effects of allowing open collaboration in establishing food sustainability [38]. It takes new ways of thinking, planning, carrying out, and cooperating, as well as the active involvement of several stakeholders from diverse sectors, to promote sustainable agriculture and nutrition [39]. Philanthropy-facilitated communication with decision-makers will aid in the growth of the food business and advance sustainability on a global scale [40].

Philanthropic organizations can be pivotal in supporting research and development by offering vital funding, resources, and expertise. Their multifaceted contributions can encompass filling financial gaps in conventional funding channels, providing seed funding for early stage research, bridge funding for promising projects requiring additional support, or sustained financing for ventures with substantial potential [41]. Embracing risk-taking with fearless resolve, philanthropic entities can champion unconventional ideas and untested hypotheses, birthing inventive solutions that redefine the nutrition and food security landscape [42]. They can further endorse interdisciplinary research, fostering collaborative efforts among experts from diverse fields to tackle complex challenges and engender innovative solutions. In addition to financial support, they can leverage their influence to champion policies and practices that foster an enabling environment for innovation, collaborating harmoniously with governments, businesses, and stakeholders [43]. Furthermore, they can advocate ardently for open science and knowledge sharing, accelerating innovation and enabling researchers to build upon each other’s work, thus contributing to the evolution of a more collaborative and efficient research ecosystem [44].

In navigating the complex terrain of nutrition and food security, philanthropic organizations, with their multifaceted contributions, stand as vital pillars in the quest for transformative change. Looking ahead, their profound role in addressing the intricate root causes of food insecurity yields key recommendations and implications for future research and practice. It is imperative to underscore the multifaceted nature of food insecurity, with interconnected root causes, demanding a comprehensive and coordinated approach [45]. Future efforts should prioritize a holistic understanding of these complexities and the development of integrated strategies for practical solutions. Several avenues hold promise for addressing the root causes of food insecurity, including investment in poverty alleviation, the promotion of sustainable agriculture through increased resource accessibility and improved farming practices, and advocacy for policy changes such as augmenting the minimum wage, broadening access to affordable healthcare, and bolstering social safety net programs [45,46,47]. 

While philanthropic endeavors to alleviate food insecurity often prioritize immediate assistance, such as food banks and emergency food aid, these interventions, while undeniably essential, tend to provide only temporary relief, potentially falling short of addressing the deep-rooted causes of food insecurity [48]. To achieve the optimal balance between immediate relief and sustainable, long-term solutions, philanthropic entities must prioritize initiatives encompassing both aspects, recognizing that addressing the underlying determinants of food insecurity is paramount. Furthermore, diversifying their intervention portfolio is essential to ensure a lasting impact. This entails investing in innovative approaches that empower communities, reduce dependency on existing food systems, and promote self-sufficiency [49,50]. By providing communities with the tools, resources, and knowledge they need to produce their own food, manage natural resources effectively, and build resilient livelihoods, we can help them become less dependent on external aid and more self-reliant. Empowering communities towards self-sufficiency in food production and livelihoods contributes to greater food security, economic stability, and the overall well-being of individuals and families (FAO 2023) [51]. It enables communities to take control of their own development trajectory, leading to more sustainable and resilient outcomes in the face of challenges such as climate change, economic shocks, and other external pressures. Exploring alternative, community-driven solutions remains integral to pursuing comprehensive food security.

In light of resource limitations, fostering collaboration and partnerships with governments, businesses, and local communities is paramount. These collaborations serve as a means to pool resources, expertise, and knowledge, ultimately maximizing the reach and effectiveness of philanthropic initiatives [52]. This collaborative approach leverages each stakeholder’s unique resources and expertise, with charitable organizations providing funding and knowledge, governments offering policy support, businesses contributing innovation and market access, and local communities supplying on-the-ground insights [52,53,54]. Such synergy leads to more potent and sustainable solutions. Collaborative efforts facilitate the scaling up of successful interventions, ensuring the adaptation of best practices to different contexts and promoting equity and inclusivity by addressing the needs of vulnerable populations [55,56].

Sustainable agriculture and nutrition are imperative for achieving a food future aligned with the SDGs and addressing the multifaceted challenges of food security. Institutional, technical, and financial breakthroughs are essential to building resilient and environmentally sustainable food systems that protect biodiversity and provide healthy diets. Philanthropic organizations are crucial in catalyzing transformative change by offering vital funding, resources, and expertise. Their contributions span diverse areas, including supporting research and development, endorsing interdisciplinary research, championing policies fostering innovation, and advocating for open science. The multifaceted nature of food insecurity requires a comprehensive and coordinated approach, prioritizing poverty alleviation, sustainable agriculture, and policy changes. Philanthropic entities such as non-governmental organizations (The World Food Programme, International Fund for Agricultural Development, Food and Agriculture Organization for the United Nations, and others) must balance immediate relief with long-term solutions, diversify their intervention portfolio, and collaborate with governments, businesses, and communities to maximize impact and promote equity. This collaborative synergy facilitates the scaling up of successful interventions and ensures the adaptation of best practices, contributing to a more potent and sustainable approach to addressing global food challenges. Furthermore, sustainable giving involves ensuring that philanthropic efforts not only address immediate needs but also work towards creating long-term solutions that empower communities to maintain their health and well-being independently. The discussion in this article aligns with the 42nd session of the FAO in October 2015 [57], where the Committee on World Food Security (CFS) convened the High Level Panel of Experts on Food Security and Nutrition (HLPE) to prepare a report on Nutrition and Food Systems. Furthermore, this topic is highly relevant to the Sustainable Development Goals (SDGs), the implementation of the Rome Declaration on Nutrition in 2014, the upcoming Decade of Action on Nutrition, and the fulfillment of the right to adequate food, aiming to analyze how food systems affect dietary patterns and nutritional outcomes of communities; and to highlight effective policies and programs that can shape food systems, contribute to improved nutrition, and ensure that food is produced, distributed, and consumed in a manner that sustains the right to adequate food for all [57]. This study is also expected to provide a set of action-oriented recommendations aimed at governments and other stakeholders as inputs for engaging researchers and philanthropists in advancing and maintaining the sustainability of nutrition and agriculture during the UN Decade of Action on Nutrition (2016–2025).

## 7. Conclusions

In conclusion, the study emphasizes the critical role of philanthropic organizations in enhancing the sustainability of agriculture and nutrition for future generations. Through aligning priorities in nutrition and public health with agricultural production, philanthropic efforts can contribute significantly to addressing challenges such as climate change, water scarcity, and food insecurity. The collaboration between philanthropic foundations, research institutions, and governmental bodies is essential for implementing sustainable practices and policies in the agricultural sector.

Furthermore, the study underscores the need for innovative approaches and financial support to drive advancements in sustainable agriculture and nutrition. Philanthropy-facilitated communication with decision-makers and stakeholders is crucial for promoting sustainable practices on a global scale. By investing in health technologies, research, and development, philanthropic organizations can play a pivotal role in shaping a more resilient and environmentally sustainable food system.

In light of the findings presented in this study, it is evident that philanthropy foundations have the potential to drive positive change in agriculture and nutrition sectors. By fostering collaboration, providing financial support, and advocating for sustainable practices, philanthropic efforts can contribute to a more secure and nutritious food future for all. It is imperative for stakeholders to continue working together towards achieving a sustainable food system that prioritizes environmental conservation, biodiversity protection, and the well-being of communities worldwide.

The urgency of addressing the root causes of food insecurity and the ethical imperative to do so in a manner that respects and promotes human dignity and rights cannot be overstated. Addressing poverty, inequality, conflict, climate change, and the lack of access to resources are crucial steps in creating sustainable solutions that uphold the fundamental rights of individuals to access adequate and nutritious food. Promoting human dignity in the context of food security involves recognizing the inherent worth and value of every individual, ensuring that they have the opportunity to lead healthy and fulfilling lives. Therefore, it is crucial to address the root causes of food insecurity in a holistic and rights-based approach, not only to alleviate immediate hunger but also to create long-term solutions that empower individuals, promote equality, and uphold their dignity and rights.

## Figures and Tables

**Table 1:** Examples of Philanthropic Initiatives Across the Food System.

Circle	Initiative	Impact
Nutrition	HarvestPlus	Increased vitamin A intake in children
	Scaling Up Nutrition (SUN) Movement	Improved nutrition outcomes in several countries
	1000 Days initiative	Improved maternal and child nutrition in developing countries
	Global Alliance for Improved Nutrition (GAIN)	Increased access to nutritious foods and improved dietary diversity
Agriculture	Gates Foundation’s Agricultural Development	Increased yields and resilience to climate change
	W.K. Kellogg Foundation’s Nourishing Territories	Enhanced biodiversity and empowered communities
	The Nature Conservancy’s Sustainable Agriculture and Water	Improved soil health and water quality, and reduced greenhouse gas emissions
	World Agroforestry Centre	Development and promotion of agroforestry practices for sustainable food production
Policy	Rockefeller Foundation’s Food Systems Vision Prize	Scaling up urban agriculture and food waste reduction initiatives
	Global Alliance for Improved Nutrition (GAIN) Advocacy Work	Improved food environments and reduced consumption of unhealthy foods
	FAO’s “SUN Movement”	Development of national nutrition plans and implementation of effective nutrition interventions
	Food and Land Use Coalition (FOLU)	Advocacy for policies that promote sustainable food and land use systems
	EAT Foundation	Development of science-based policy recommendations for sustainable food systems

## Data Availability

There are no data contained in this opinion article, and the data were only sourced from the literature listed in this article.
